# Characterisation of a novel p53 mutation (p53_N263Tfs*7_) associated with li-fraumeni syndrome

**DOI:** 10.1186/s12885-026-16582-2

**Published:** 2026-07-29

**Authors:** Francesca M. Wright, Mariela Vasileva-Slaveva, Angel Yordanov, Ivan Ivanov, Zornitsa Gorcheva, Andrea Mohr, Gareth S. A. Wright, Greg N. Brooke, Metodi V. Metodiev

**Affiliations:** 1 School of Life Sciences, University of Essex, Colchester, Essex CO4 3SQ UK; 2 Department of Breast Surgery, Shterev Hospital, Sofia, 1000 Bulgaria; 3 Bulgarian Breast and other gynecological Cancers Association, Sofia, 1750 Bulgaria; 4 Medical University of Pleven, Pleven, 5800 Bulgaria

**Keywords:** TP53, Lee-Fraumeni Syndrome, Mutations, Breast cancer

## Abstract

Li-Fraumeni Syndrome (LFS) is a hereditary cancer predisposition syndrome caused by germline mutations in the *TP53* tumour suppressor gene, which encodes the multifunctional transcription factor p53. p53 is the most commonly mutated protein in human cancer, with the majority occurring within the DNA-binding domain, often disrupting transcriptional activity and resulting in a loss-of-function. Here, we characterise the novel p53_N263Tfs*7_ truncated mutant, identified as a germline mutation from a patient with LFS who developed breast cancer. Functional assays revealed a partial loss-of-function across key cellular processes, including proliferation, cell death, cell motility, and transcriptional transactivation. This mutant lacked a dominant-negative effect, distinguishing it from common DNA-binding domain missense mutations. Our findings demonstrate that oncogenesis in LFS can be driven by partial impairment of functional p53, rather than dominant-negative or gain-of-function mutations alone. This underscores the clinical significance of recognising subtle *TP53* variants for the refined molecular classification and clinical prediction of cancer risk in *TP53* mutation carriers.

**Supplementary Information:**

The online version contains supplementary material available at https://doi.org/10.1186/s12885-026-16582-2.

## Background

In 1969, Frederick Li and Joseph Fraumeni described four families in which multiple children had developed soft-tissue sarcomas, with early-onset cancers also observed in at least one young adult within the family [1]. As a result, they suggested a hereditary syndrome of cancer predisposition [2], which is now known as Li-Fraumeni Syndrome (LFS). It is now understood that the majority of families (*∼* 70–80%) meeting the classic LFS criteria carry a pathogenic germline *TP53* mutation [3–5]. *TP53* is a tumour suppressor gene that plays a pivotal role in maintaining genomic integrity and cellular homeostasis [6]. Its encoded protein, p53, is regulated by post-translational modifications, such as phosphorylation, acetylation, and ubiquitination [7]. Stabilising post-translational modifications generally occur in response to various cellular stresses, including DNA damage, hypoxia, and oncogene activation [8]. This stabilisation and activation of p53 allows it to carry out functions in a variety of different processes, including DNA repair mechanisms, programmed cell death pathways (such as apoptosis and autophagy) [9], and the induction of cell cycle arrest and cellular senescence [7].

*TP53* is the most frequently mutated gene in human cancers [10, 11], with the majority occurring within the DNA-binding domain (DBD) [12]. This pattern is replicated in LFS-associated *TP53* mutations, as missense mutations in the DBD comprise the vast majority of germline *TP53* mutations [3]. As a consequence of mutations, p53’s binding to downstream response elements (REs) is compromised, often leading to loss-of-function (LOF) [13]. Further to this observed LOF, gain-of-function mutations (GOF), which confer oncogenic activity onto the mutant p53 protein, have also been suggested [14]. This aspect of p53 mutations is a controversial topic, and a wealth of literature exists both accepting and refuting claims of GOF p53 mutations [12].

The dominant-negative activity of specific p53 mutations is a well-recognised mechanism of tumour suppression loss [15]. Given that the majority of p53 mutations are found within the DBD, mutants often retain the capacity to oligomerise, forming functional tetrameric complexes, and it has subsequently been demonstrated that wild-type and mutant p53 proteins can form inhibitory heterotetramer complexes [16]. In instances where dominant-negative effects (DNE) are active, mutant subunits within the heterotetramer may induce conformational changes in wild-type subunits and function is subsequently abrogated [15]. Further, the extent of the DNE appears to correlate with the number of mutant p53 subunits in the heterotetramer complex [17]. DNEs often play a critical role in the oncogenesis of p53 heterozygous tumours, due to the ability to attenuate the tumour-suppressing functions of wild-type p53 through heterotetramer complex formation [12].

Importantly, not all *TP53* mutations are expected to behave equivalently. While the majority of cancer-associated and LFS-associated *TP53* variants are missense mutations within the DBD that retain an intact tetramerisation domain, a smaller subset consists of truncating variants that result in loss of all or part of the C-terminus. Studies have demonstrated substantial heterogeneity among *TP53* variants, with missense mutants frequently exhibiting dominant-negative activity and, in some cases, GOF properties, whereas truncating variants are generally associated with loss of protein function and reduced capacity to interfere with wild-type p53 [18, 19]. Consequently, the functional consequences of *TP53* truncating mutations cannot be inferred solely from studies of common DNA-binding domain missense mutants.

Despite its low prevalence, LFS is highly penetrant, such that carriers of germline *TP53* mutations have a 50% probability of developing cancer by age 30 [20], increasing to a 90% probability within the lifetime of the carrier [21]. This compares to germline *BRCA1/BRCA2* mutations, which are significantly more common among the general population, but carry a lower risk of breast cancer development than germline *TP53* mutations [22]. Furthermore, it has been shown that penetrance is highly dependent on both the cancer tissue type and the gender of the carrier [23].

In this study, a novel p53 mutation identified from a patient diagnosed with LFS, and a paternal history of cancer was characterised. This patient presented with osteosarcoma of the knee at an early age and subsequently developed breast cancer at 27. The mutation in question consists of a 5 bp deletion in exon 8 (NM_000546.6:c.788_792del), resulting in a frameshift and the introduction of a premature stop codon 7 amino acids downstream, at position 269. This truncated protein (NP_000537.3:p.(N263Tfs*7)), now referred to as N263Tfs*7, lacks the C-terminus, which houses the tetramerisation domain (TD). To elucidate the functional consequences of this truncation, we performed a series of experiments; comparing our mutant to both wild-type p53 and a transcriptionally inactive control mutant, C135Y [24]. Overall, our findings indicate that the N263Tfs*7 mutation is hypomorphic, resulting in severely compromised tumour suppressive activity across proliferation, cell death, motility, and transcriptional transactivation. By characterising p53_N263Tfs*7_, we demonstrate that a partial, quantitative reduction in functional p53 levels can be sufficient to drive oncogenesis in the context of LFS, highlighting the need for a more nuanced molecular classification of *TP53* variants, as recognising these subtle impairments may support more accurate clinical risk prediction and management in mutation carriers.

## Methods

### 3D modelling of p53_N263Tfs*7_

Protein domain maps were prepared for p53_WT_, p53_C135Y_, and p53_N263Tfs*7_ using the drawProteins package [25] in R v4.3.2 [26]. Invasive breast carcinoma datasets from cBioPortal [27–29], were compiled and used to identify p53 mutations. The amino acid sequence for p53_N263Tfs*7_ was used to predict the 3-dimensional structure of the mutant protein using AlphaFold3 [30]. The predicted structure, and the 4HJE wild-type tetrameric crystal structure of the p53 core domain in the presence of DNA [31] were compared using PyMol v3.1.6.1 [32].

### Site-directed mutagenesis

GFP-tagged p53_WT_ was synthesised and cloned into a pTWIST expression vector (pTWIST-GFP-p53_WT_). Mutagenesis primers for p53_C135Y_ and p53_N263Tfs*7_ were designed using the Agilent QuikChange Primer Design tool, and ordered from IDT (Table 1). Mutagenesis was performed using an Agilent QuikChange II Site-Directed Mutagenesis Kit according to the manufacturer’s instructions, with the following modification: 2.9% (v/v) DMSO was also added to each mutagenesis reaction. Successful mutagenesis was confirmed using Sanger sequencing.

### Cell culture

Cells were acquired from ATCC, and frozen at a low passage. Cells were cultured in Dulbecco’s Modified Eagle’s Medium, supplemented with 10% (v/v) Fetal Bovine Serum. Cells were passaged twice weekly. Fresh aliquots were defrosted every six months.

### Western blotting

HeLa cells were grown to 70–90% confluency in a 6-well plate and transfected with 4 *μ*g of pTWIST-GFP constructs using TurboFect, following the manufacturer’s instructions. Cells were harvested using a cell scraper and pelleted (1,500 rpm for 3 min). The supernatant was aspirated and the cells washed with 1 ml PBS. Cells were re-pelleted (1,500 rpm for 3 min), the supernatant aspirated, and the pellet lysed in RIPA buffer. Western blotting was performed as described [33], with the following modifications: 40 *μ*g of protein was separated, and electrophoretic transfer onto nitrocellulose membranes was performed using the iBlot™ 2 Gel Transfer Device. Primary antibodies used were; GFP B-2 (Santa Cruz Biotechnology, sc-9996), p53 (Dako, M3629), and *α*-tubulin (Proteintech, 11224-1-AP). HRP-conjugated secondary antibodies used were; anti-mouse (Proteintech, SA00001-1), and anti-rabbit (Proteintech, SA00001-2). Densitometry quantifications were performed using ImageJ 1.54g [34].

### Fluorescence imaging

HeLa cells were seeded onto glass coverslips at a seeding density of 5*×*10^5^ cells/ml in a 12-well plate. Cells were transfected with 2 *μ*g of pTWIST-GFP constructs using TurboFect, following the manufacturer’s instructions, and incubated for 24 h. Cells were fixed in 2% w/v PFA at room temperature for 10 min, washed with PBS and mounted on slides using DAPI mountant (ProLong™ Gold Antifade Mountant, Invitrogen). Images were acquired using an EVOS M5000 Imaging System. Image analysis was performed using ImageJ [34], to separate and quantify the nuclear and cytoplasmic GFP signals.

### Subcellular protein fractionation

HEK293S cells were grown to 70–90% confluency in a 6-well plate and transfected with 4 *μ*g of plasmid using TurboFect. Subcellular protein fractionation was performed using a Subcellular Protein Fractionation Kit for Cultured Cells (Thermo Scientific), following the manufacturer’s instructions. Protein localisation was visualised by Western blotting and probing with anti-GFP antibody (Santa Cruz Biotechnology, sc-9996), as described (Western blotting).

### Luciferase reporter assay

HeLa cells were grown to 70–90% confluency in a 24-well plate and transfected with 200 ng pTWIST-GFP constructs, 200 ng p21luc plasmid, and 100 ng BOS-*β*-Gal plasmid using a calcium phosphate transfection method [35]. 24 h after transfection, cells were washed with PBS and fresh media added. Cells were incubated for a further 24 h before lysis and subsequent luciferase and *β*-galactosidase activities were measured, as described [36]. Luciferase activity was normalised to *β*-galactosidase activity, and expressed as a percentage of pTWIST-GFP-p53_WT_ activity.

### Quantitative PCR analysis of gene expression

HeLa cells were seeded in 12 well plates, transfected with 1 *μ*g of pTWIST-GFP-p53 construct using Turbofect and incubated for 48 h prior to collection. RNA extraction and qPCR was performed as previously described [37] and gene expression normalised to transfection efficiency (% GFP positive cells). Primer sequences (5’−3’): *L19 *F – GCGGAAGGGTACAGCCAAT, *L19 *R – GCAGCCGGCGCAAA; *p21* F – GACACCACTGGAGGGTGACT; *p21* R – CAGGTCCACATGGTCTTCCT; *BAX* F – CCCGAGAGGTCCTTTTCCG, BAX R – GCACTCCCGCCACAAAGAT.

### Caspase-GLO 3/7 assay

HEK293T cells were grown to 70–90% confluency in a 6-well plate and transfected with 4 *μ*g pTWIST-GFP constructs using TurboFect. Transfected cells were sub-cultured into 96-well plates at a seeding density of 3.6*×*10^4^ cells/ml. Cells were incubated for 72 hours before imaging using a SARTORIOUS Incucyte S3 to quantify the number of GFP-positive cells. Caspase-GLO 3/7 assay was performed according to the manufacturer’s instructions. The luminescence for each pTWIST-GFP construct was normalised to the corresponding number of GFP-positive cells and expressed as a percentage of caspase-3/7 activity in pTWIST-GFP (Empty) control.

### Growth assay

HEK293T cells were grown to 70–90% confluency in a 6-well plate and transfected with 4 *μ*g pTWIST-GFP constructs using TurboFect. Transfected cells were sub-cultured into a 24-well plate at a seeding density of 6*×*10^4^ cells/ml. Images were acquired in six-hour intervals, using a SARTORIUS Incucyte S3, for three days.

### Wound healing assay

HEK293T cells were grown to 70–90% confluency in a 6-well plate and transfected with 4 *μ*g pTWIST-GFP constructs using TurboFect. Using a P10 micropipette tip, “wounds” were scratched into the bottom of each transfected well. Media was aspirated, cells were washed with PBS, and fresh media added. Images were acquired in four-hour intervals, using a SARTORIUS Incucyte S3, for two days. Images were exported and the area of the scratch analysed in ImageJ [34], using Manual Wound Healing Size Tool for stack analysis [38].

### CRISPR-select^TIME^ assay

HEK293T cells were grown to 70–90% confluency in a 24-well plate. 1 *μ*g of donor DNA (the p53_N263Tfs*7_ mutant or a synonymous WT control, p53_SYN_, used for internal normalisation; Table 2) was transfected into cells using Lipofectamine CRISPRMAX Reagent (Thermo Scientific) RNP method, with 1 *μ*g purified Cas9 protein and 250 ng gRNA (Table 2). Donor DNA and gRNA were designed using Invitrogen TrueDesign Genome Editor [39]. For mixing of cell populations, cells were trypsinised, resuspended in media and centrifuged (2000 rpm, 4 min). The pellets were resuspended in media, the cell suspensions combined, and split equally into a 24-well plate. For each condition, two independent CRISPR editing/transfection experiments were performed. Following transfection, cells from the two independent experiments were combined and seeded as a single mixed cell population for subsequent harvesting and analysis.

For harvesting of mixed cell populations, cells were trypsinised, resuspended in media and centrifuged (2000 rpm, 4 min). The pellets were frozen at −70 $^{\circ }$C until further processing. Cells were harvested in 24-hour intervals. Genomic DNA was isolated using 0.2 M NaOH and regions of interest were amplified using PCR amplification (Table 2); Q5 High-Fidelity DNA Polymerase (New England BioLabs). Products were separated using agarose gel electrophoresis, and purified using a GeneJET Gel Extraction and DNA Cleanup Micro Kit (Thermo Scientific). The DNA concentration was measured using a NanoDrop spectrophotometer, and samples sent to GENEWIZ UK Ltd for NGS. FASTQ files were subsequently analysed using MutScan v1.14.1 [40], by defining the left flanking sequence, the specified mutation, and the right flanking sequence for each CRISPR-induced mutation (Table 3).

### Statistical analyses

For the fluorescence microscopy image analysis and qPCR, one-way ANOVA and post-hoc (Tukey’s HSD) tests were performed using R v4.3.2 [26]. For the luciferase reporter and Caspase-GLO 3/7 assays one-tailed heteroscedastic t-tests were performed. For the growth and wound healing assays, ANOVA and post-hoc (Tukey’s HSD) tests were performed using R v4.3.2 [26], and Bonferroni correction was applied.

## Results

### p53_N263Tfs*7_ leads to a premature stop codon in the DNA binding domain

**Fig. 1:**
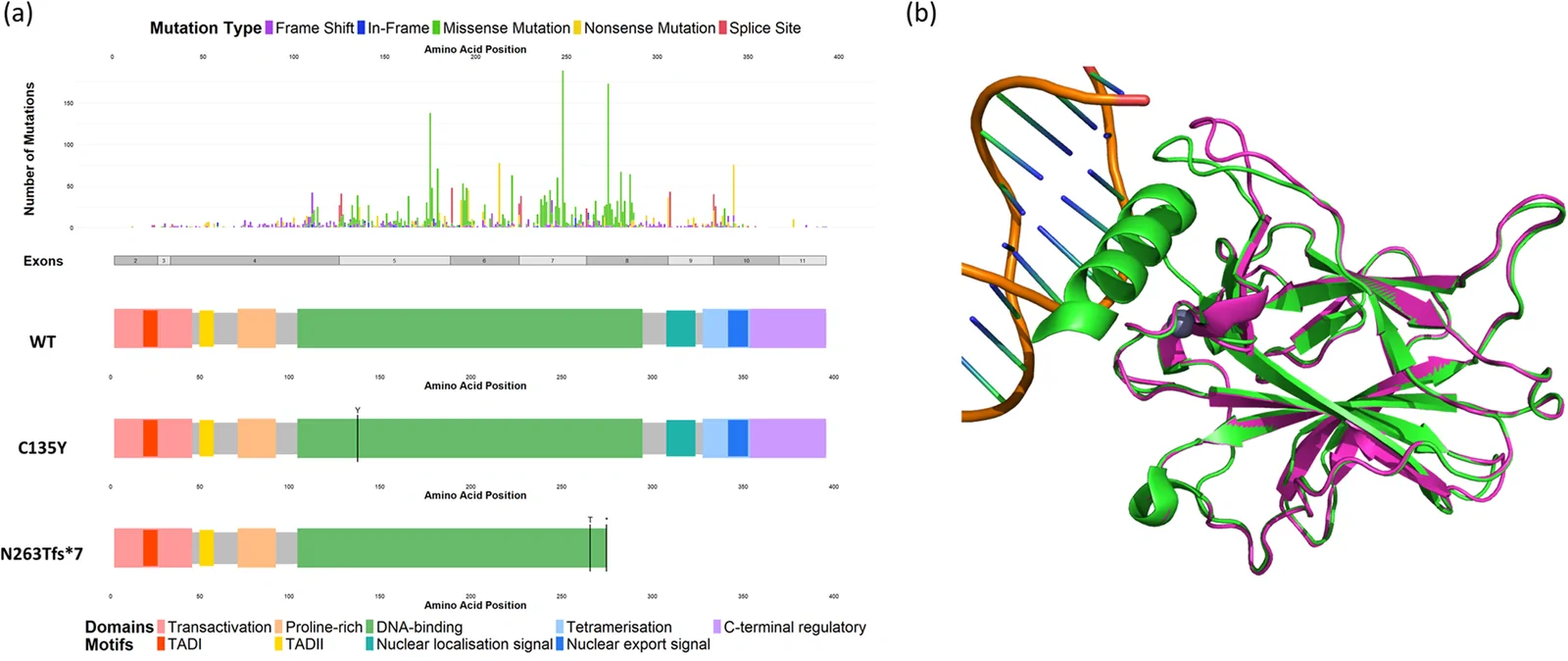
Analysis of p53 mutations in breast cancer and the structure of p53_N263Tfs*7_. **a** Analysis of p53 mutations by amino acid residue in breast invasive carcinoma was performed, and mapped to exons and domains of p53. The location of the p53_C135Y_ point mutation and p53_N263Tfs*7_ deletion start site are marked with solid black lines and the new amino acids indicated, while the p53_N263Tfs*7_ premature stop codon is marked with a dashed black line. **b** 3-dimensional predictive modelling of p53_N263Tfs*7_ was performed using AlphaFold3 (magenta) and compared to the 4HJE crystal structure of p53_WT_ (green) using PyMol

A novel p53 mutation was identified in a patient with LFS. The patient was originally diagnosed with sarcoma and then developed breast cancer. Sequencing analysis of the individual’s blood sample identified a heterozygous germline p53 mutation that consisted of a deletion of ATCTA starting at Asn263. The resulting frameshift introduces six new amino acids (Thr-Gly-Thr-Glu-Gln-Leu) before a premature stop codon at position 269. The truncated protein subsequently lacks the 3’ end of the DBD, the TD, and the C-terminal regulatory domain, as well as the nuclear localisation signal (NLS) and the nuclear export signal (NES). To better understand if this region of p53 is commonly mutated in breast cancer, the cBioPortal repository was interrogated [27–29]. Missense mutations in the DBD were the most frequently observed alteration, while 15% of the patient samples analysed exhibited truncations (Fig. 1a). The asparagine residue at position 263 is rarely mutated in breast cancer, occurring in 0.08% of the analysed patient samples.

To investigate the potential effect of the truncation upon p53, AlphaFold3 was used to predict the 3-dimensional structure of p53_N263Tfs*7_, and the resulting structure was overlaid with the 4HJE crystal structure of the p53_WT_ core domain (Fig. 1b). The p53_N263Tfs*7_ predicted structure aligns well with p53_WT_, with ordered regions exhibiting greater consensus than disordered regions, with the *α*-helix at residues 273–291 completely lost. This suggests that DNA binding may be reduced as a result of the truncation, regardless of consensus between the 4HJE crystal structure of the p53_WT_ core domain and the AlphaFold3 predicted structure of p53_N263Tfs*7_.

### p53_N263Tfs*7_ results in protein truncation

**Fig. 2:**
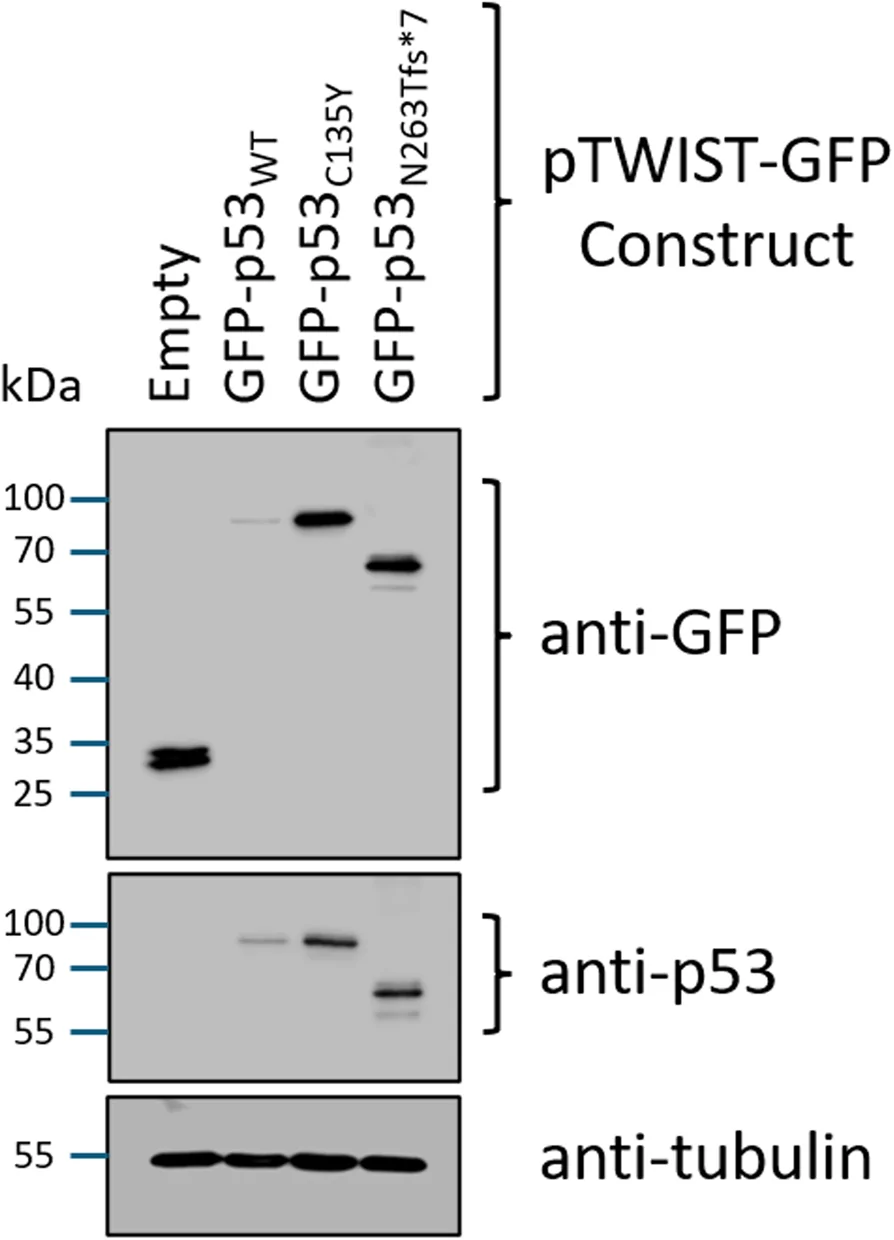
The p53_N263Tfs*7_ mutation increases protein abundance in HeLa cells. HeLa cells were grown and transfected with N-terminally GFP-tagged p53 constructs. Equal amounts of protein from each transfected culture were subjected to western blot analysis using anti-GFP and anti-p53. Tubulin was used as a loading control. Representative of two independent repeats

To generate N-terminally GFP-tagged p53-mutant constructs, site-directed mutagenesis was performed on pTWIST-GFP-p53_WT_ and successful mutagenesis confirmed using Sanger sequencing (Supplementary Table 8). To confirm expression of wild-type and mutant p53, HeLa cells were transfected with N-terminally GFP-tagged p53 constructs and immunoblotting was performed (Fig. 2). As expected, p53_WT_ and p53_C135Y_ were approximately 90 kDa in size (p53 + *∼*27 kDa GFP). The truncation caused by p53_N263Tfs*7_ should result in a protein of approximately 36 kDa, which with the GFP-tag should give a fusion protein of 63 kDa, in agreement with what was observed on the western blot. Relative p53_C135Y/N263Tfs*7_ expression was 28.4- and 28.7-fold higher than p53_WT_, respectively, suggesting that these mutants are resistant to the proteasomal degradation which regulates p53_WT_ abundance [41].

### p53_N263Tfs*7_ has altered cellular localisation compared to p53_WT_

**Fig. 3:**
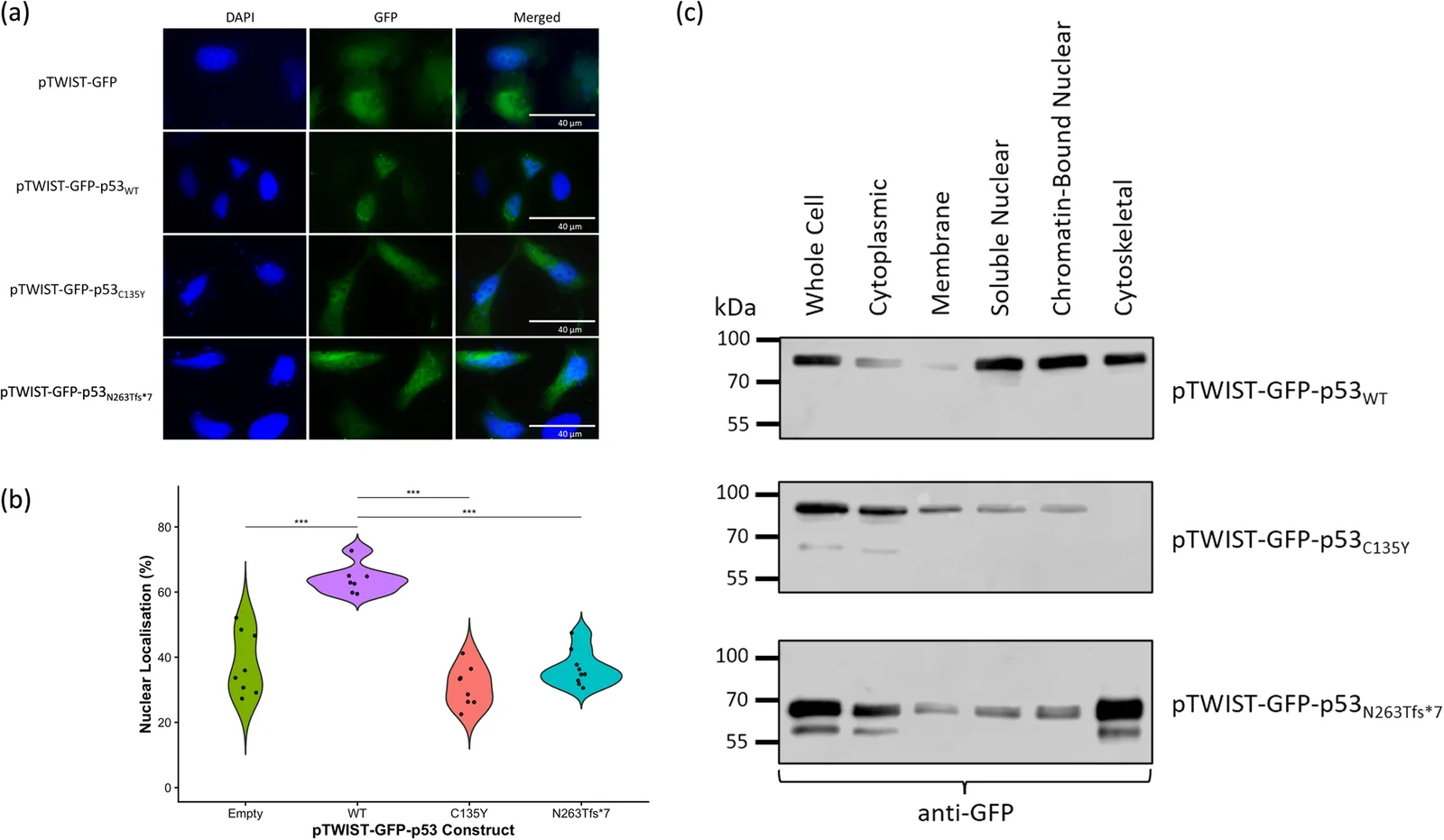
The p53_N263Tfs*7_ mutation reduces nuclear localisation of the protein. **a** HeLa cells grown on coverslips were transfected with pTWIST-GFP constructs. The subcellular localisation of p53 was determined by staining cells with DAPI mountant and fluorescence imaging, and **b** the nuclear localisation was quantified using ImageJ. *** *p < 0.001*. Localisation analysis was performed on a minimum of 7 cells per construct. **c** HEK293S cells were grown and transfected with pTWIST-GFP-p53 constructs. Proteins from all fractions were subjected to western blotting using anti-GFP antibody. Representative images from 2 independent experiments

To analyse the localisation of p53_N263Tfs*7_, HeLa cells were transfected with GFP-tagged constructs and imaged using fluorescent microscopy. pTWIST-GFP was observed to localise diffusely throughout the cell, p53_WT_ appeared to predominantly localise to the nuclear compartment (63.9%), while p53_C135Y/N263Tfs*7_ were observed to predominantly localise to the cytoplasmic compartment (31.0% and 36.5% respectively) (Fig. 3a, b). To further analyse the subcellular localisation of p53, cell fractionation was performed. p53_WT_ was found to be predominantly nuclear and chromatin-bound. A significant fraction was also found to be associated with the cytoskeleton. In agreement with the imaging results, p53_C135Y_ was less nuclear than p53_WT_. p53_N263Tfs*7_ had a similar distribution to p53_C135Y_, although there was a significant portion associated with the cytoskeleton (Fig. 3c). Both mutants therefore have reduced nuclear localisation and reduced chromatin binding.

### p53_N263Tfs*7_ has reduced transcriptional activity

**Fig. 4:**
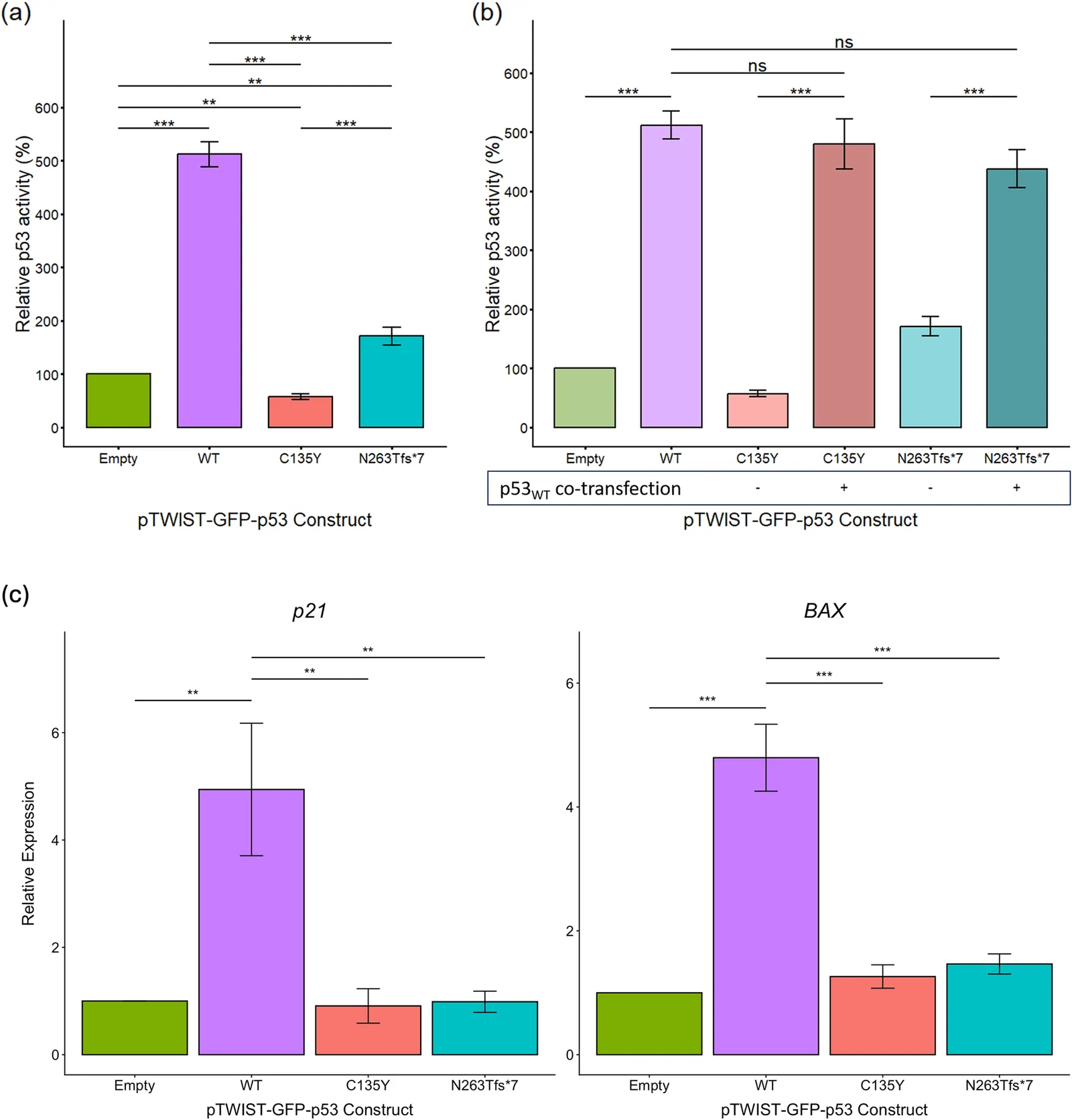
The p53_N263Tfs*7_ mutation reduces transcriptional transactivation of p53 REs. HeLa cells were grown and co-transfected with p21luc and BOS-*β*-gal plasmids and **a** 200 ng of pTWIST-GFP constructs or **b** plasmid DNA totalling 400 ng consisting of combinations of either pTWIST-GFP or pTWIST-GFP-p53_WT_, with pTWIST-GFP-p53_C135Y/N263Tfs*7_ constructs. Cells were lysed and subjected to luciferase reporter assays to measure reporter activation and infer activity of p53 mutants. Mean of three independent repeats with duplicates ± SE. t-test ** *p < 0.01*, *** *p < 0.001*, ns = not significant. **c** HeLa cells were grown and transfected with pTWIST-GFP constructs. RNA extraction and qPCR were performed to assess relative expression of *p21* and *BAX* mRNA. Mean of four independent repeats ± SE. ANOVA and Tukey’s HSD ** *p < 0.01*, *** *< 0.0001*

To analyse the transcriptional activity of p53_N263Tfs*7_, HeLa cells were co-transfected with the GFP-tagged constructs, p21luc and BOS-*β*-gal plasmids, and luciferase reporter assays performed (Fig. 4a). p53_WT_ increased reporter activity 51-fold compared to basal levels. p53_C135Y_ was found to be transcriptionally inactive, whereas p53_N263Tfs*7_ retained a small amount of transcriptional activity, increasing reporter activity 1.7-fold. As mentioned previously, some p53 mutants appear to have dominant-negative activity over p53_WT_. To determine if p53_N263Tfs*7_ is able to exert dominant-negative or wild-type rescue effects, reporter assays were repeated, with cells co-transfected with pTWIST-GFP-p53_WT_ ± -GFP-p53_C135Y/N263Tfs*7_ (Fig. 4b). Co-transfection with the mutant constructs (at a 1:1:1 molar ratio, p53_WT_:p53_N263Tfs*7_:p21luc) had no significant effect on p53_WT_ activity. Neither of the mutants were therefore found to exert DNE over p53_WT_.

To investigate the effect of the mutants on endogenous gene expression, the levels of the established p53 target genes *p21* and *BAX* were quantified. HeLa cells were transfected with the p53 plasmids or empty control and incubated for 48 h prior to harvesting for qPCR analysis (Fig. 4c). As expected, p53_WT_ significantly increased the expression of both target genes. In contrast, neither mutant altered target gene expression. Although p53_N263Tfs*7_ appeared to increase *BAX* expression (approximately 1.5-fold), this did not reach significance.

### p53_N263Tfs*7_ has reduced inhibitory activity upon cell proliferation

**Fig. 5:**
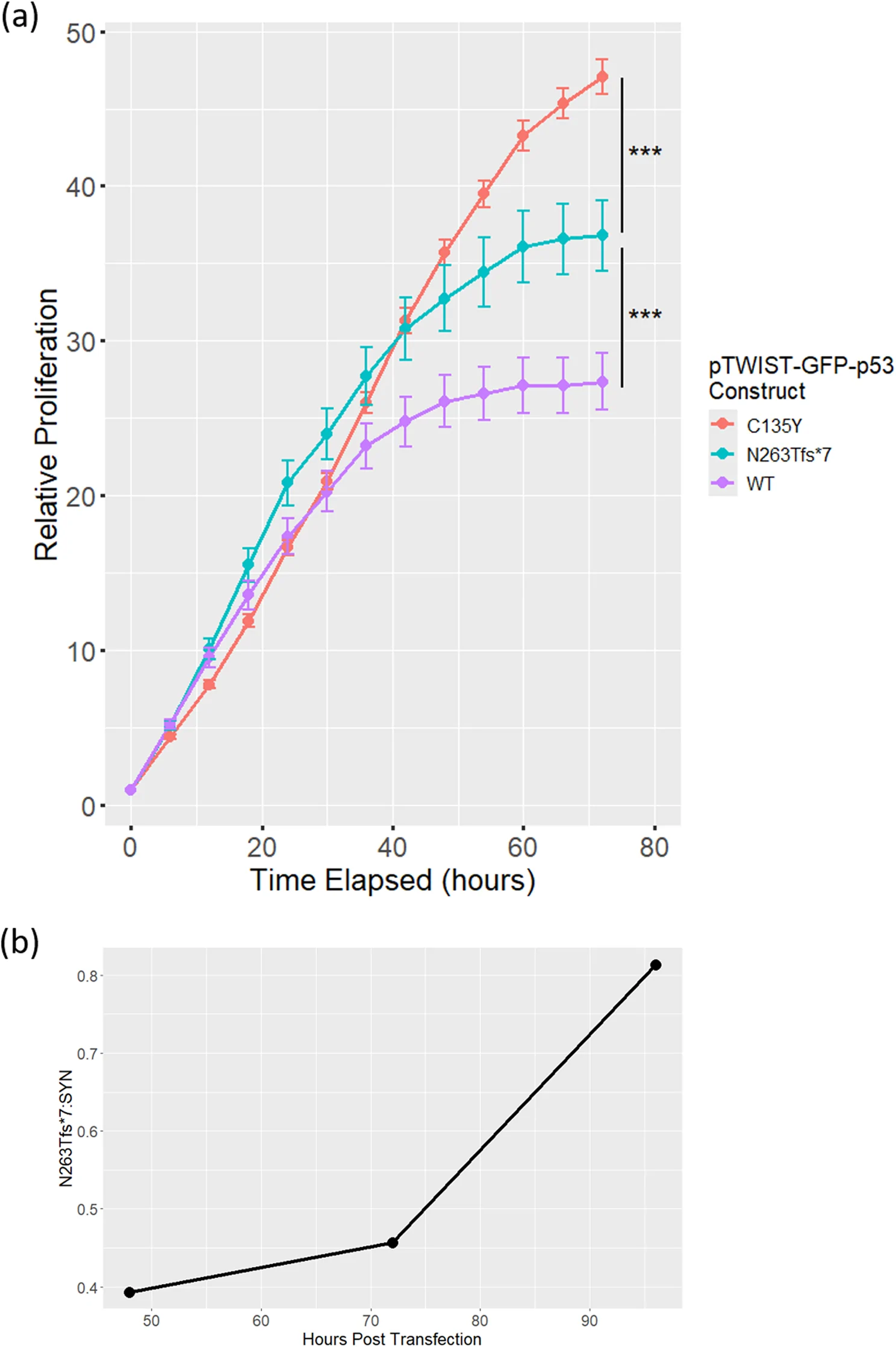
The p53_N263Tfs*7_ mutation has reduced inhibitory effect upon proliferation. **a** HEK293T cells were grown and transfected with pTWIST-GFP-p53 constructs, and seeded at a low density into a 24-well plate. The total number of green cells were counted and made relative to timepoint 0. A two-way ANOVA was performed, followed by post-hoc testing with Bonferroni correction. Mean of 3 independent repeats, with 6 wells per condition and 4 images taken per well ± SE. ANOVA and Tukey’s HSD *** *< 0.0001*. **b** HEK293T cells were subjected to CRISPR-Select^TIME^ assays. For each condition, two independent CRISPR editing/transfection experiments were performed and pooled prior to initiation of the time course. Amplicon NGS was performed on samples collected at 24 h intervals, and the ratio of p53_N263Tfs*7_ to WT-synonymous mutation (p53_SYN_) reads calculated

To assess the effect of p53 mutants on proliferation, growth assays were performed. HEK293T cells were transfected with GFP-tagged p53 constructs and images were taken every six hours for a total of 72 hours (Fig. 5a). Cells expressing p53_C135Y_ remained in the exponential growth phase longer than cells expressing p53_WT/N263Tfs*7_, with growth beginning to plateau at *∼*60 hours. Conversely, cells expressing p53_WT_ plateaued considerably earlier, at *∼*30 hours, while cells expressing p53_N263Tfs*7_ plateaued at an intermediate time point. The p53_N263Tfs*7_ mutation therefore has reduced tumour suppressive activity compared to p53_WT_.

To investigate the effect of p53_N263Tfs*7_ upon proliferation further, HEK293T cells were subjected to CRISPR-Select^TIME^ assays [42]. Mixed cell populations, containing p53_N263Tfs*7_ edited cells and cells containing the WT-synonymous mutation (p53_SYN_), were harvested at 24-hour intervals. The region surrounding the edited bases (*∼*500 bp) was PCR amplified, and the resulting DNA sent for NGS. FASTQ files were analysed using MutScan [40], to determine the relative number of mutant reads over time. The ratio of p53_N263Tfs*7_ edited cells to p53_SYN_ edited cells increased 2.1-fold over the duration of the assay (Fig. 5b). The CRISPR-Select^TIME^ assay data are therefore consistent with a selective advantage of p53_N263Tfs*7_ relative to the p53_SYN_ control, providing supporting evidence that the p53 variant confers proliferative advantages over p53_WT_.

### The p53_N263Tfs*7_ truncation impairs pro-apoptotic signalling

**Fig. 6:**
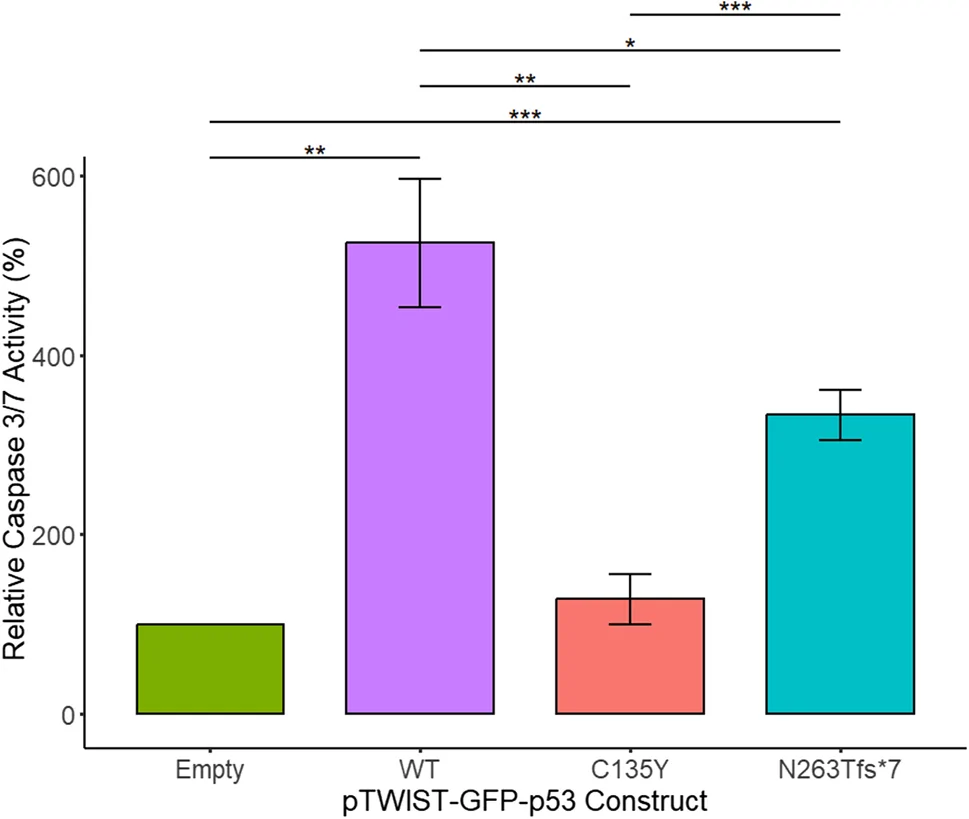
The p53_N263Tfs*7_ mutation reduces caspase 3/7 apoptotic activity. HEK293T cells were grown and transfected with pTWIST-GFP constructs, and grown in a 24-well plate. Cells were mixed with Caspase-GLO 3/7 reagent, and the luminescent signal measured to show the relative apoptosis induction of p53 mutants. Mean of three independent repeats with duplicates ± SE. t-test * p < 0.05** *p < 0.01*, *** *p < 0.001*

As N263Tfs*7 reduced the inhibitory effects of p53 upon proliferation and motility, we were interested to see if this truncation also has an impact upon cell death. To analyse the apoptotic caspase activity of p53 mutants, HEK293T cells were transfected with pTWIST-GFP-p53 constructs and Caspase-3/7 assays performed. p53_WT_ increased caspase 3/7 activity 5.3-fold compared to basal levels. Conversely, p53_C135Y_ resulted in a non-significant 1.3-fold increase. Interestingly, p53_N263Tfs*7_ increased caspase 3/7 activity 3.3-fold, and this was found to be significantly lower than p53_WT_ (Fig. 6). p53_N263Tfs*7_ therefore has reduced pro-apoptotic activity compared to p53_WT_, although some activity is retained.

### The p53_N263Tfs*7_ mutation inhibits anti-metastatic tumour suppressive functions

**Fig. 7:**
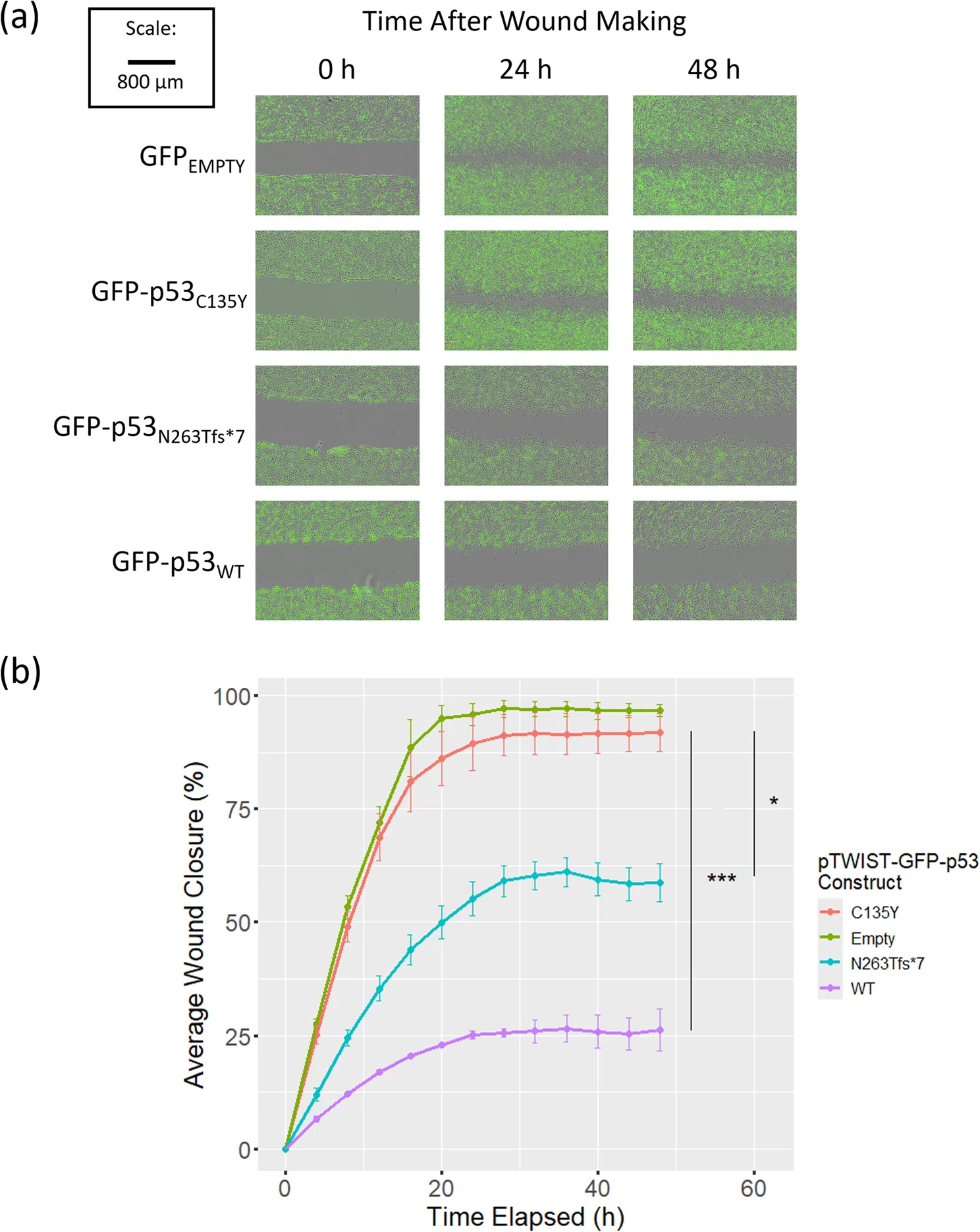
The p53_N263Tfs*7_ mutation increases the migratory capacity of cells in culture. HEK293T cells were grown and transfected with pTWSIT-GFP constructs, and wounds scratched into the bottom of each well. (A) Images were acquired every 4 hours using SARTORIUS Incucyte S3, and (B) the area of the wound at each time point was analysed using Manual Wound Healing Size Tool for stack analysis in ImageJ. One-way ANOVA and Tukey’s HSD analyses were performed, with Bonferroni correction. Mean of 3 independent repeats, with 3 images taken per well ± SE. ANOVA and Tukey’s HSD * *p < 0.05*, *** *p < 0.0001*

p53 mutants are typically associated with invasive and metastatic cancers, suggesting a role for p53 in cell migration [43]. To assess the effect of p53 mutants on migration, HEK293T cells were transfected with the p53 constructs. “Wounds” were introduced into the cell monolayer, and images taken every 4 hours (Fig. 7a). After 48 hours, both pTWIST-GFP and p53_C135Y_ exhibited almost complete wound closure, while p53_WT_ showed very low levels of wound closure. p53_N263Tfs*7_ showed intermediate levels of wound closure, significantly different from p53_WT/C135Y_ (Fig. 7b). Over the 48-hour period, p53_WT_ and p53_N263Tfs*7_ also showed a decrease in monolayer density (Fig. 7a). As with previous experiments, p53_N263Tfs*7_ therefore has reduced, but not completely impaired, tumour suppressor activity.

## Discussion

This study investigated the functional implications of a novel germline p53 mutation identified in a blood sample from a breast cancer patient diagnosed with LFS and a previous osteosarcoma diagnosis. p53 is a well-studied protein, with considerable literature dissecting its involvement in several key mechanisms and pathways. Our aim was to determine the functional consequences associated with this novel mutation, and we subsequently demonstrated that p53_N263Tfs*7_ is a hypomorphic LOF mutant that retains residual transcriptional and tumour-suppressive activity, distinctly lacking DNE upon p53_WT_.

Mutations in the DBD of p53 are well-documented, representing the most common alteration in p53 [44]. Analysis of cBioPortal breast invasive carcinoma datasets [27–29], found that mutations at residue 263 are rare, accounting for *∼*0.08% of mutations. This translates to only three reported mutations in this location, all of which were frame shift mutations. The frame shift associated with p53_N263Tfs*7_ results in the introduction of a stop codon at position 269.

To assess the potential structural basis for observed functional defects, the 3D conformation of p53_N263Tfs*7_ was predicted and compared to the crystal structure of wild-type p53 in the presence of DNA [31]. The model did not show significant conformational deviations from the wild-type structure, despite lacking the H2 *α*-helix at residues 278–292 [45]. The H2 helix sits in the DNA major groove [46], allowing A276, C277 and R280 to form hydrogen bonds and hydrophobic interactions at the major groove [47]. The R273 residue, which is also deleted in the p53_N263Tfs*7_ mutant, is also critical for DNA binding at the major groove, due to a direct interaction with the DNA backbone [47]. Mutations at R273 are common in cancer, with the residue classed as a “hotspot” for cancer-associated mutations [48], which are known to abrogate DNA binding [49]. Therefore, the p53_N263Tfs*7_ deletion leaves fewer of the critical DNA-binding residues intact, suggesting that interaction with DNA will be significantly attenuated, consistent with the tempered transcriptional activity observed in the reporter assays.

p53’s tumour suppressor functions are critically dependent on its activation via post-translational modifications and subsequent nuclear translocation [50]. However, many p53 mutants exhibit altered subcellular localisation patterns, with notable aberrant cytoplasmic sequestration observed in various tumour types [51]. This phenomenon is often correlated with poor prognosis, and poor responses to chemotherapy [52, 53], prompting investigations into its underlying mechanisms. In the context of our study, localisation experiments revealed that a large proportion of p53_N263Tfs*7_ was extranuclear. Specifically, we observed that p53_N263Tfs*7_ exhibited a predominantly cytoplasmic distribution, accompanied by a noticeable reduction in nuclear (both soluble and chromatin-bound) accumulation compared to p53_WT_. This finding aligns with previous reports of cytoplasmic sequestration for other p53 mutants. The cytoplasmic retention of mutant p53, as observed for N263Tfs*7, could stem from several factors. Given that N263Tfs*7 involves a truncation that excludes residues 269–393, encompassing the 3’ end of the DBD, the TD, and the C-terminal domain, including the NLSs and NES [54]. It is therefore likely that the altered localisation is due to the lack of the NLSs [55], with conformational changes, the presumed inability to tetramerise [56], and a possible increased affinity for cytoskeletal filament systems [57], contributing to its cytoplasmic sequestration.

While p53 predominantly exerts tumour suppressive functions through its role as a transcription factor, mislocalisation to the cytoplasm is significant as p53 has also been shown to play a role in cytoplasmic functions, including mitochondrial-driven apoptosis [58], metabolism [59], and autophagy [60]. If a substantial portion of the p53 protein is sequestered in the cytoplasm, it logically follows that a lower concentration of p53 would be present in the nucleus. This reduced nuclear presence offers a plausible explanation for the impaired function observed for p53_N263Tfs*7_, as it is unable to access its nuclear targets [61]. However p53_N263Tfs*7_ does show some nuclear localisation, supporting the reduced activity [62]. This differential localisation thus provides a key mechanism underpinning the varied functional outcomes observed among different p53 mutants.

p53 exerts its tumour suppressive functions primarily as a transcription factor, binding to specific DNA REs within the promoters of its target genes to regulate their expression [50, 63]. This leads to the induction of genes involved in cell cycle arrest, apoptosis, DNA repair, and senescence [64]. A common consequence of mutations in the p53 DBD is a loss of this critical transcriptional transactivation capability, thereby abrogating its tumour suppressor function [65, 66]. In the context of our study, luciferase reporter assays and qPCR provided clear evidence that p53_N263Tfs*7_ exhibits significantly impaired transcriptional transactivation activity compared to p53_WT_. Specifically, we observed a profound reduction in the ability of p53_N263Tfs*7_ to activate *p21* and *BAX* transcription.

The observed deficiency in transcriptional activity for p53_N263Tfs*7_ can be attributed to several mechanisms, many of which are interconnected. Foremost, its aberrant cytoplasmic sequestration would inherently limit the amount of protein able to reach the nucleus and interact with its DNA targets [61]. Beyond localisation defects, tetramerisation is often considered essential for the transcriptional activity of p53. Given that the p53_N263Tfs*7_ does not possess a TD, the subsequent lack of tetramerisation is likely to impair the transcriptional activity of the p53 proteins that are localised to the nucleus [56]. The low-level of activity of p53_N263Tfs*7_ observed in the reporter assays, may be driven by the ability of monomeric p53 units to bind their REs, albeit with a *∼*100-fold lower affinity compared to the functional homotetramer [67]. Importantly, this low level of activity, did not translate into significant induction of the endogenous p53 target genes *p21* or *BAX*. This apparent discrepancy may reflect the greater sensitivity of reporter assays, or suggest that the residual transcriptional activity of p53_N263Tfs*7_ is insufficient to induce robust expression of endogenous target genes. Nevertheless, p53_N263Tfs*7_ retained partial tumour suppressive activity in the proliferation, apoptosis and migration assays. It is therefore possible that these residual biological effects are mediated through low-level regulation of alternative p53 target genes that were not examined in this study and/or through transcription-independent functions of p53. Indeed, p53 has been shown to promote apoptosis directly at the mitochondria through interactions with BCL-2 family proteins, independently of its role as a transcription factor [68]. Given the predominantly cytoplasmic localisation of p53_N263Tfs*7_, such mechanisms may contribute to the residual functional activity observed in our assays.

Co-transfection experiments further elucidated the functional characteristics of p53_N263Tfs*7_. The behaviour of p53_N263Tfs*7_ highlights an important distinction between truncating *TP53* variants and the more extensively studied missense mutations within the DNA-binding domain. Large-scale genotype-phenotype studies have shown that many missense mutants retain the ability to oligomerise with wild-type p53 and frequently exert dominant-negative effects that contribute substantially to pathogenicity [18, 19]. In contrast, truncating variants that remove the tetramerisation domain are predicted to be functionally distinct because they are unlikely to form heterotetramers with wild-type p53. Consistent with this model, co-transfection experiments demonstrated that p53_N263Tfs*7_ did not inhibit the transcriptional activity of wild-type p53, indicating an absence of detectable dominant-negative activity. Rather than actively suppressing the remaining wild-type allele, p53_N263Tfs*7_ appears to contribute to carcinogenesis through a quantitative reduction in functional p53 activity. The reduced transcriptional activity, impaired induction of p53 target genes, and diminished effects on proliferation, apoptosis and migration observed throughout this study support a hypomorphic loss-of-function phenotype. These findings add to growing evidence that *TP53* variants exist along a functional spectrum, ranging from dominant-negative and gain-of-function missense mutants to truncating alleles that retain limited residual tumour suppressor activity.

A limitation of this study is the use of HeLa and HEK293-derived cell lines for functional characterisation. Both cell types express viral proteins that are known to interact with the p53 pathway. In HeLa cells, the HPV E6 oncoprotein promotes p53 degradation [69], while adenoviral proteins expressed in HEK293 cells can modulate p53 stability and transcriptional responses [70]. These interactions may influence the magnitude of p53-dependent phenotypes observed in our assays. Nevertheless, all constructs were evaluated within the same cellular background, allowing direct comparison of relative differences between the p53 variants. Future studies using p53-null cellular models, would provide a cleaner genetic background in which to further define the intrinsic functional properties of p53_N263Tfs*7_ and validate the observations reported here.

In summary, our comprehensive functional characterisation of the novel LFS associated p53_N263Tfs*7_ mutant reveals a hypomorphic LOF phenotype, consistently demonstrated across localisation, transcriptional activity, cell growth, death and migration assays. We found that p53_N263Tfs*7_ exhibits aberrant cytoplasmic sequestration due to the loss of key nuclear import/export signals, which significantly reduces its nuclear localisation. This mislocalisation, alongside the presumed structural inability to tetramerise, underpins the observed phenotype: a profound impairment of tumour suppressive functions, including transcriptional transactivation, and proliferative, apoptotic and migratory capacities. The absence of DNE on p53_WT_ suggests that p53_N263Tfs*7_ behaves differently from many canonical DBD missense mutants, operating primarily as a hypomorphic LOF allele rather than an active inhibitor of wild-type p53. The distinct functional profile is consistent with a loss of tumour suppressor activity where the quantitative reduction in effective p53 is sufficient to drive disease. Specifically, the reduced nuclear concentration of functional p53 promotes aggressive cellular behaviour, contributing to increased proliferation and potential metastatic progression in the context of inherited cancer predisposition. Our findings demonstrate that oncogenesis in LFS can arise from partial impairments in p53 activity, rather than solely through dominant-negative or GOF mutations. Consequently, a more refined molecular classification of *TP53* variants that accounts for these hypomorphic LOF mutations could support more accurate predictions of cancer risk and disease progression in clinical practice.

## Supplementary Information

Supplementary Material 1.

## Figures and Tables

**Fig. 8:**
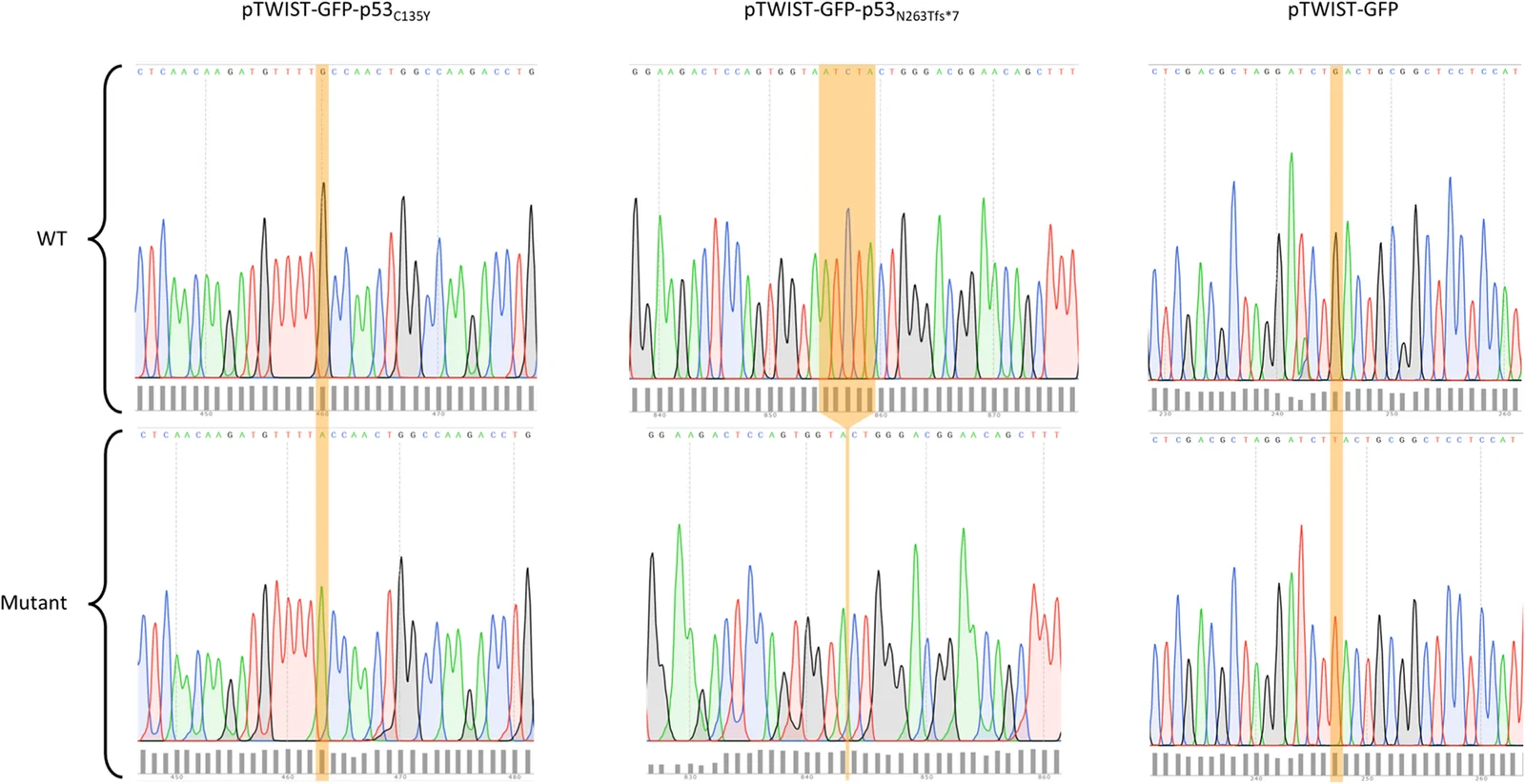
Successful mutagenesis of pGFP-WT p53. Site-directed mutagenesis was performed using an Agilent QuikChange II Site-Directed Mutagenesis Kit, and successful mutagenesis was confirmed via sequencing. Chromatograms show successful the point mutations for pTWIST-GFP-p53_C135Y_ and pTWIST-GFP, and the successful deletion for pTWIST-GFP-p53_p.N263Tfs*7_

**Fig. 9:**
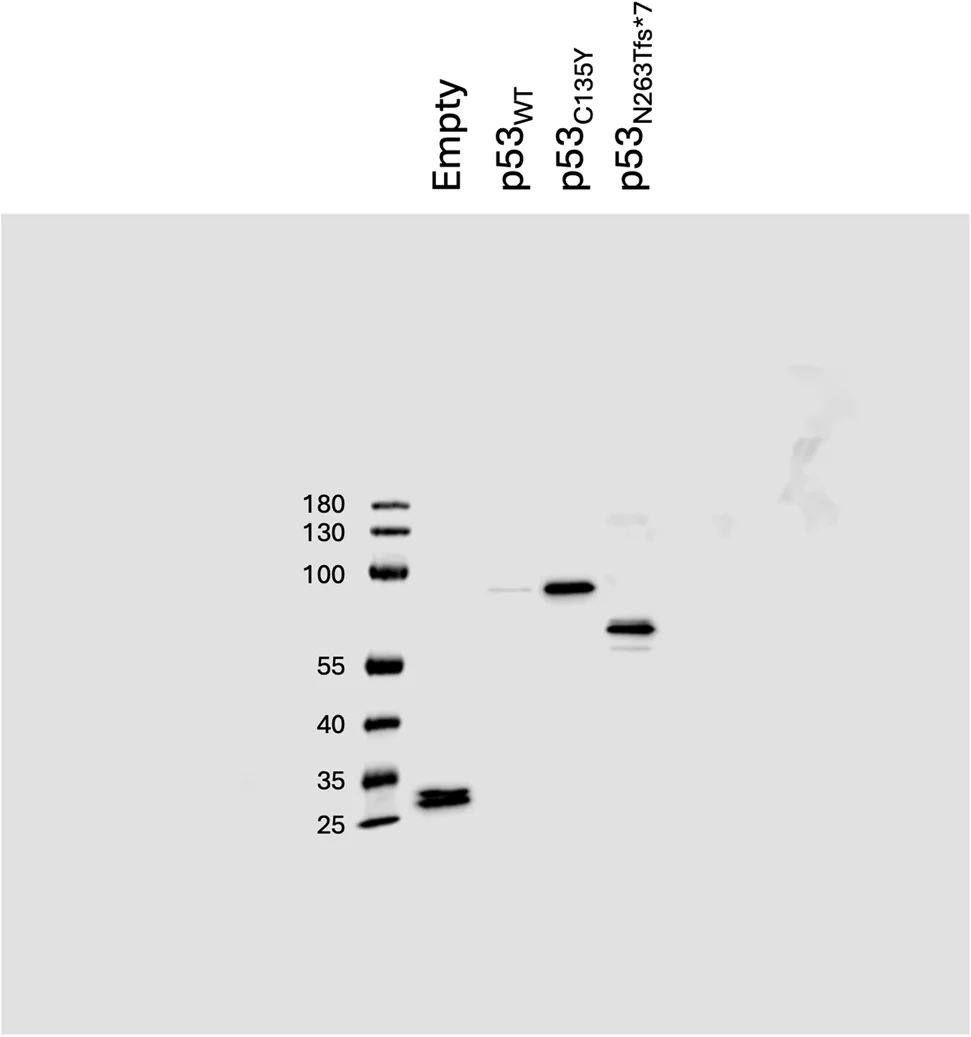
Chemiluminescent detection of pTWIST-GFP-p53 constructs with anti-GFP. Unprocessed blots as presented in the manuscript Fig. 2

**Fig. 10:**
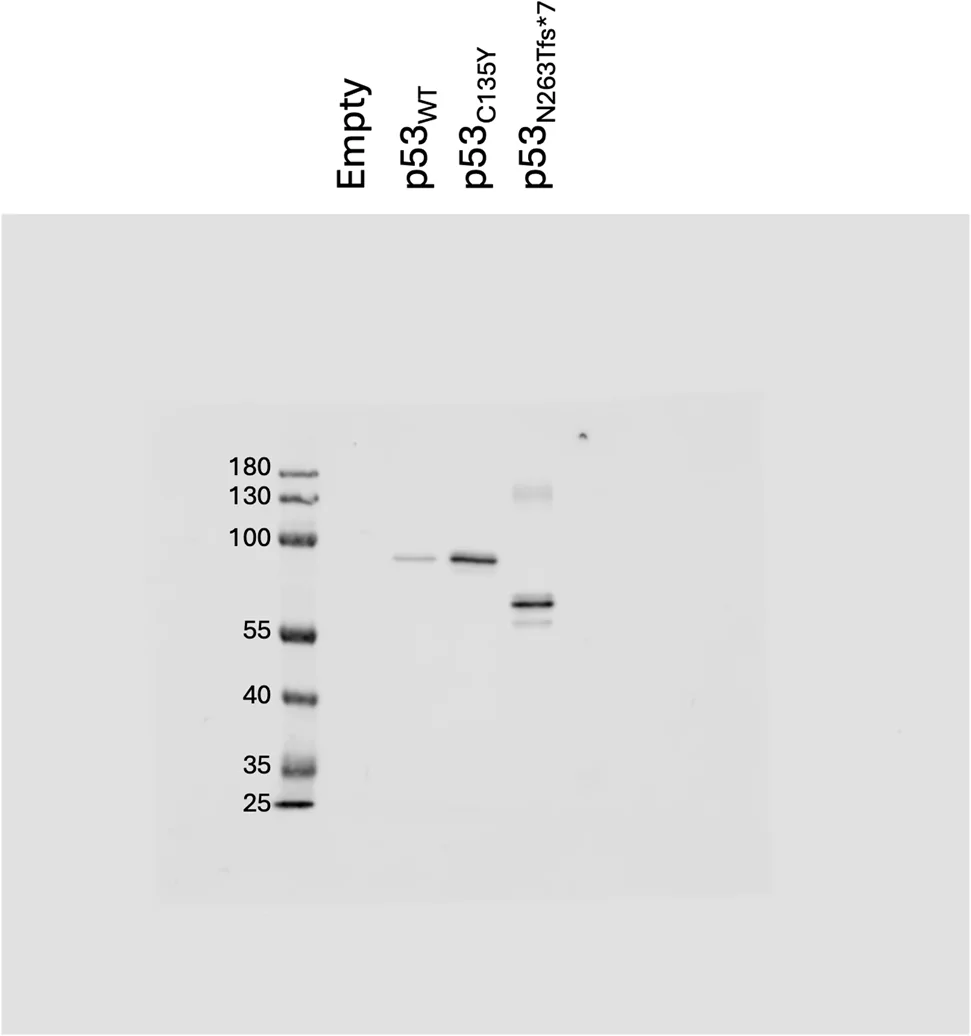
Chemiluminescent detection of pTWIST-GFP-p53 constructs with anti-p53. Unprocessed blots as presented in the manuscript Fig. 2

**Fig. 11:**
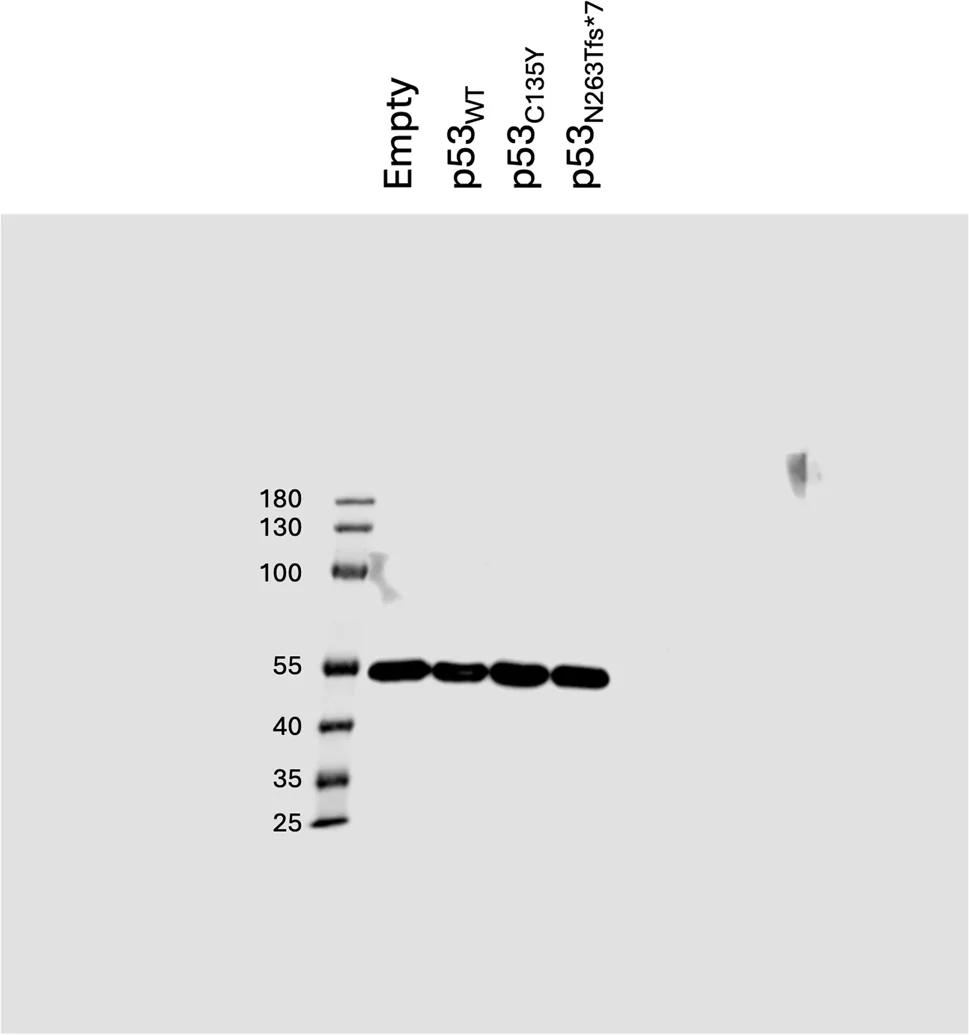
Chemiluminescent detection of Tubulin. Unprocessed blots as presented in the manuscript Fig. 2

**Fig. 12:**
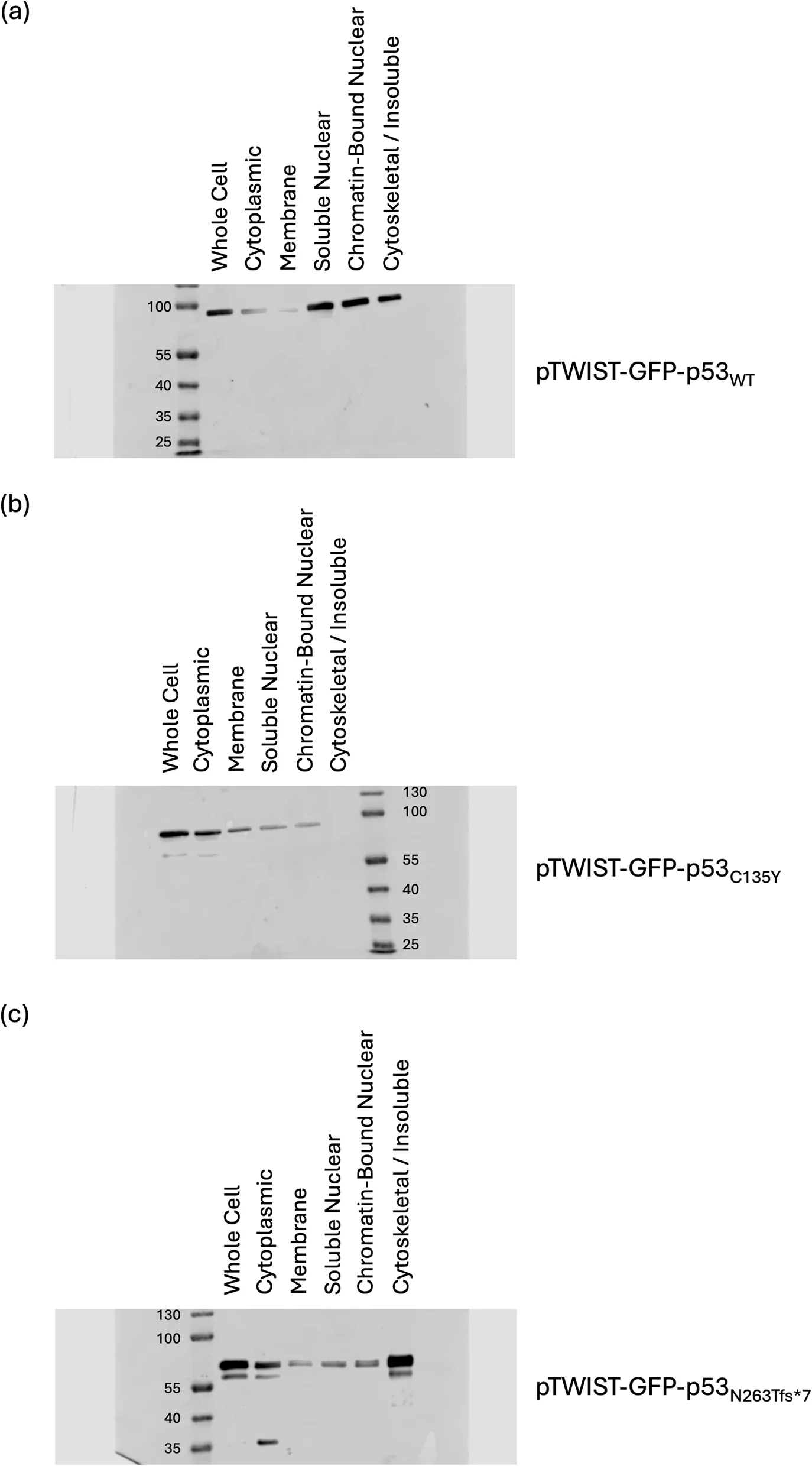
Chemiluminescent detection of pTWIST-GFP-p53 constructs with anti-GFP after subcellular fractionation. Unprocessed blots for detection of **a** p53_WT_, **b** p53_C135Y_, and **c** p53_N263Tfs*7_ as presented in the manuscript Fig. 3B

## Data Availability

All data supporting the findings of this study are available within the paper and its Supplementary Information. Primer sequences and donor DNA sequences used in the CRISPR-Select experiments are provided in Supplementary Tables A1 and A2.

## Appendix A

### Sequence information

**Table 1:** Forward and reverse primer sequences for site-directed mutagenesis of pTWIST-GFP-p53

Primer	Sequence
pTWIST-GFP-p53_C135Y_ forward	5’-CCCTGCCCTCAACAAGATGTTTTACCAACTGGCCAA-3’
pTWIST-GFP-p53_C135Y_ reverse	5’-TTGGCCAGTTGGTAAAACATCTTGTTGAGGGCAGGG-3’
pTWIST-GFP-p53_N263Tfs*7_ forward	5’-ACTGGAAGACTCCAGTGGTACTGGGACGGAAC-3’
pTWIST-GFP-p53_N263Tfs*7_ reverse	5’-GTTCCGTCCCAGTACCACTGGAGTCTTCCAGT-3’
pTWIST-GFP forward	5’-ATGGAGGAGCCGCAGTAAGATCCTAGCGTC-3’
pTWIST-GFP reverse	5’-GACGCTAGGATCTTACTGCGGCTCCTCCAT-3’

**Table 2:** Sequences for components of the CRISPR-Select^TIME^ assay

Component	Sequence
p53_N263Tfs*7_ Donor DNA	5’-CCTCTTGCTTCTCTTTTCCTATCCTGAGTAGTGGTACTGG GACGGAACAGCTTTGAGGTGCGTGTTTGTG-3’
p53_SYN_ Donor DNA	5’-TCTTGCTTCTCTTTTCCTATCCTGAGTAGTGGTAACCTAC TGGGACGGAACAGCTTTGAGGTGCGTGTTTGT-3’
gRNA	5’-CCTGAGTAGTGGTAATCTAC-3’
PCR forward primer	5’-ACACTCTTTCCCTACACGACGCTCTTCCGATCTCCATACT ACTACCCATCCACC-3’
PCR reverse primer	5’-GACTGGAGTTCAGACGTGTGCTCTTCCGATCTCTTGTCC TGCTTGCTTACC-3’

**Table 3:** MutScan Analysis of FASTQ Files. For each mutation, the left flanking sequence, specified mutation, and right flanking sequence were defined. MutScan was then used to identify the number of reads for each of the specified mutations

Left flanking sequence	Specified mutation	Right flanking sequence
**N263Tfs*7**		
CCTCTTGCTTCTCTTTTCCTATCCTGAGTAGTGGTA		CTGGGACGGAACAGCTTTGAGGTGCGTGTTTGTG
**SYN**		
CCTCTTGCTTCTCTTTTCCTATCCTGAGTAGTGGTAA	C	CTACTGGGACGGAACAGCTTTGAGGTGCGTGTTTGTG

## Abbreviations

DBD: DNA binding domain
DNE: Dominant-negative effects
GOF: Gain-of-function
LOF: Loss-of-function
NES: Nuclear export signal
NGS: Next generation sequencing
NLS: Nuclear localisation signal
REs: Response elements
SYN: Synonymous WT control
TD: Tetramerisation domain
