# Supplementary material for: Characterisation of a novel p53 mutation (p53N263Tfs*7) associated with li-fraumeni syndrome
Source: BMC Cancer. 2026 Jul 29;26:1297. doi: 10.1186/s12885-026-16582-2 (PMC13700178; doi:10.1186/s12885-026-16582-2)
Supplement: Supplementary file 1 [file 12885_2026_16582_MOESM1_ESM.zip › Wright et al. Uncropped blots.pdf]

Uncropped blots for Figure 2

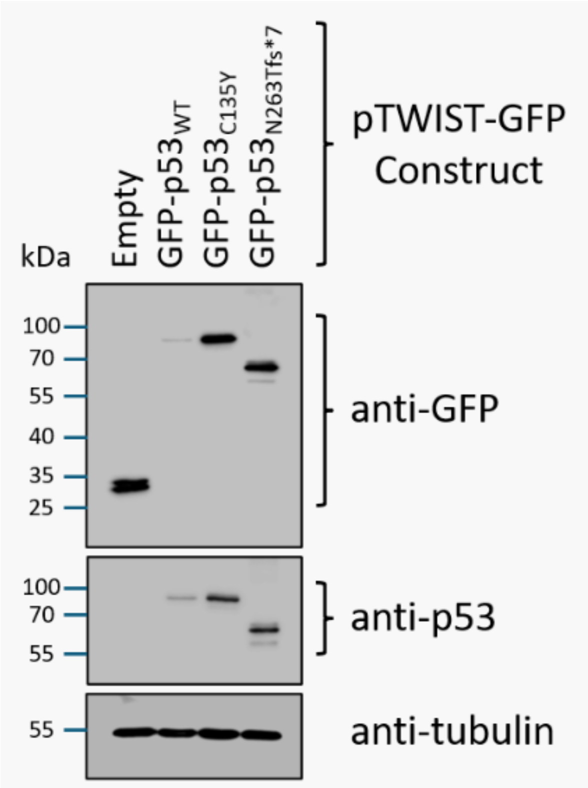

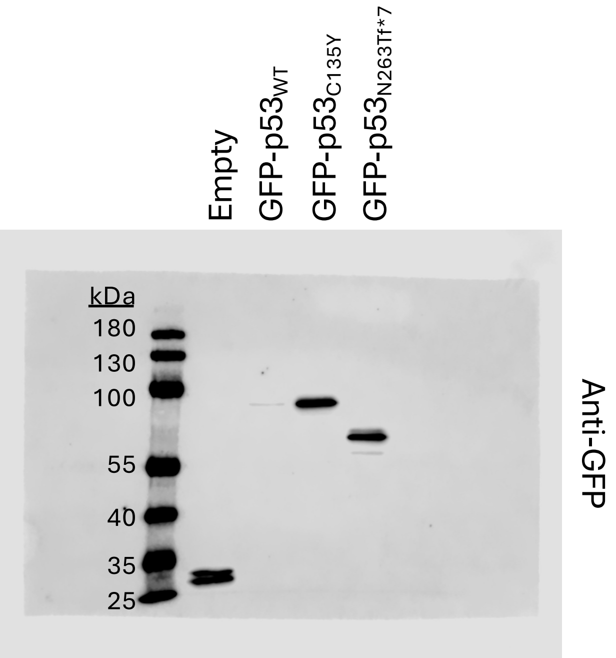

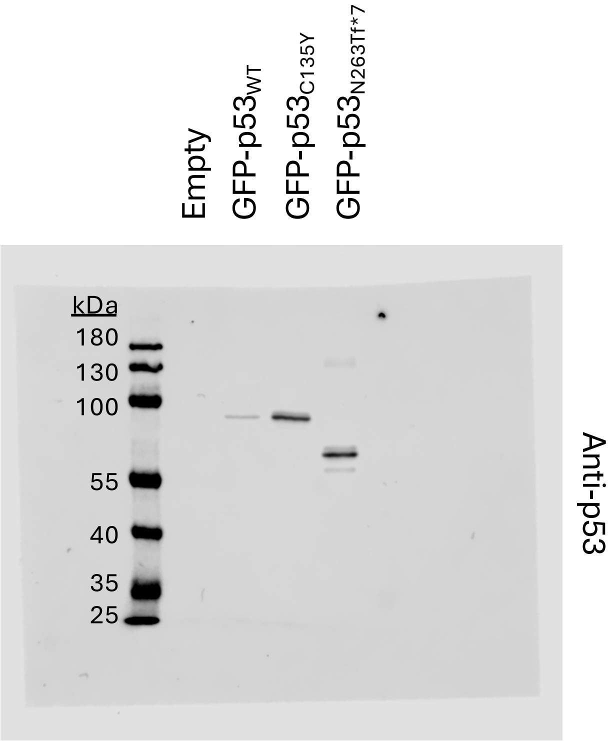

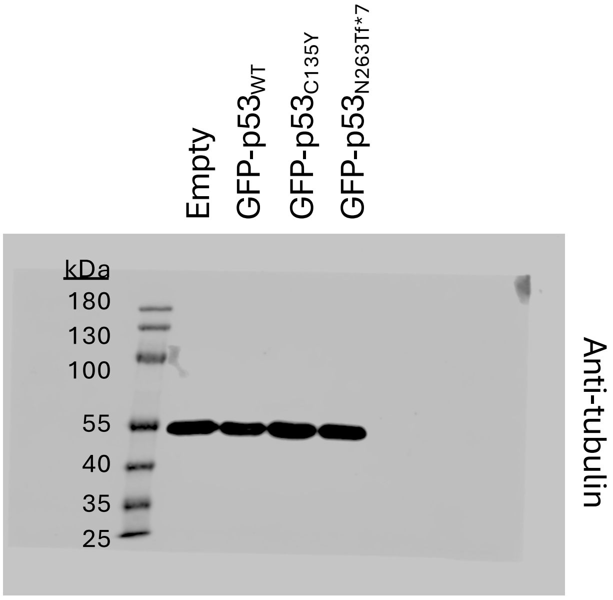

Uncropped blots for Figure 3b

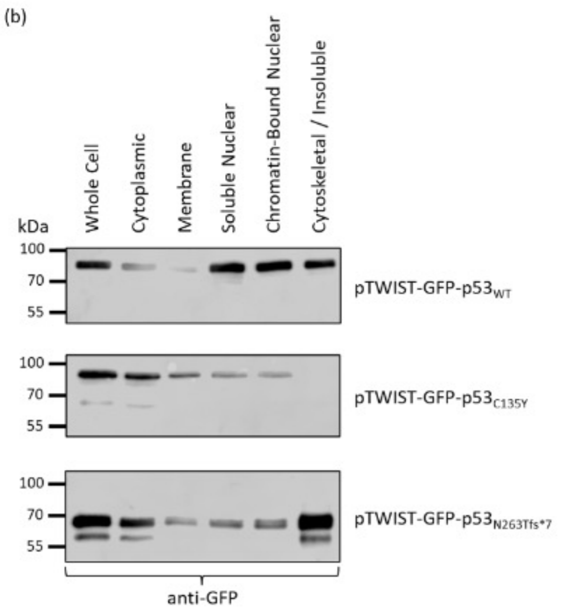

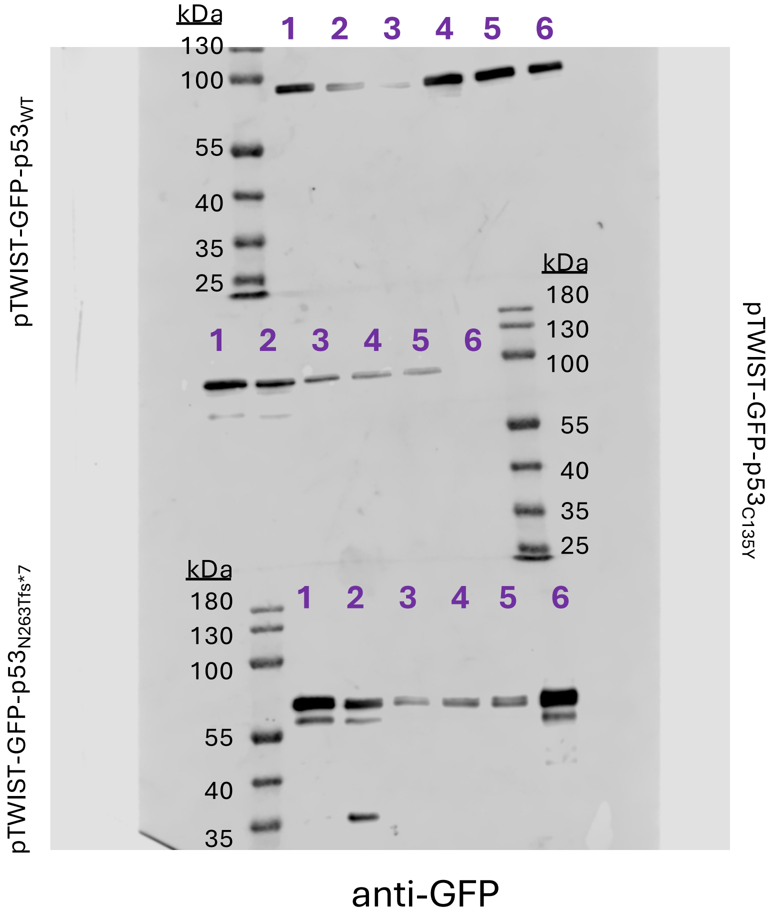

Lanes

1 = Whole cell
2 = Cytoplasmic
3 = Membrane
4 = Soluble Nuclear
5 = Chromatin-Bound Nuclear
6 = Cytoskeleton / Insoluble
